# Heightened protein-translation activities in mammalian cells and the disease/treatment implications

**DOI:** 10.1093/nsr/nwaa066

**Published:** 2020-04-14

**Authors:** Chung-I Wu, Haijun Wen

**Affiliations:** State Key Laboratory of Biocontrol, School of Life Sciences, Sun Yat-sen University, China; State Key Laboratory of Biocontrol, School of Life Sciences, Sun Yat-sen University, China

After cells divide, the new cells have to synthesize all the necessary cellular components in time for the next division. There is a lower bound of time required for cells to double their contents. For mammalian cells, this lower bound may be around 20 hours, as artificial selection for faster-dividing cells has not pushed cell lines to go below this ‘barrier’ in doubling time. The occasional exceptions are those that appear to ‘prove the rule’. For example, yeast cells can divide once every 1.5 hours [1,2], and the fastest rate of cell divisions in metazoans may be that of the embryonic cells of *Drosophila*, at 5 minutes per cycle [3]. Thus, at least for eukaryotic cells, the rate-limiting step is not the replication of DNA.

This rate-limiting step is central to cell biology as well as many diseases (although emphasis has been shifting to mutation-based approaches such as gene-targeting and vaccine development). For the components along the central dogma, each cell has only two copies of the DNA for every gene and the median number of mRNAs in mammalian cells has been reported to be ∼17. Strikingly, the median number of proteins is 50 000 [4]. As there is a 3000-fold increase in quantity from mRNA to protein, whereas the increases in DNA and mRNA content are only 2-fold and 17-fold, respectively, the rate-limiting step is likely to be protein synthesis [5]. Indeed, protein translation is an energy-demanding process [6], consuming 30% of the energy used by mammalian cells.

Normal cells apparently function within the constraint imposed by the rate-limiting step. Similarly, the limit may not pose a hurdle for most disease progressions as cells of the diseased tissues usually under-perform without exceeding the limit. However, there are two types of cells that may push close to, or even beyond, the limit; these cells ‘over-perform’ in the translation step.

The first type is cancer cells, which divide much more often than normal cells, thus they have to translate proteins at a much higher rate than normal cells. The second type is cells infected with viruses, which force their ‘host’ cells to make large quantities of proteins. Here, controlling the rate-limiting step may hold the key to dampening protein translation and, hence, alleviating symptoms or even suppressing disease. At the present time, virus-infected cells deserve intense attention. We will nevertheless discuss cancer cells briefly in terms of conceptual background and resource availability, which are better understood in cancer than in virus infection.

## CANCERS

In the last two decades, cancer research has focused on the mutations underlying tumorigenesis, with efforts culminating in the search for ‘driver mutations’ (TCGA [7–9]). The literature is massive but a simple lesson has been that different tumors, even of the same pathological type, often have non-overlapping sets of mutated genes [7,8,10]. Tumors are, hence, like unhappy families, each being unhappy in its own way. The divergent genetic bases driving tumorigenesis are reflected in the low reproducibility of many cancer biological studies [11–13]. Different studies may encounter different evolutionary trajectories that do not repeat themselves [14,15].

Furthermore, for those cancer cases with known driver mutations, gene-targeting treatments have not worked out as well as expected because of within-tumor heterogeneity [16–18]. For example, Ling *et al*. [17] showed that the basic neutral evolutionary process leads to massive accumulations of coding region mutations, even in tumors of moderate size. Because almost all coding site mutations are expected in such tumors, the emergence of resistance to targeting therapy is anticipated.

Against this backdrop, the more traditional chemotherapies that target cell components involved in tumorigenesis deserve renewed attention. Chemotherapies do target molecules that function in some aspects of cell proliferation, but are only weakly affected by somatic mutations. In other words, they target the molecular ‘phenotypes’ rather than the genetic mutations. Using chemo-agents, it may be most effective to focus on the rate-limiting step in cell division, which is likely to be protein translation (as described previously). Targeting the translation step quantitatively can be done by i) reducing the output of ribosomes (including rRNA and ribo-proteins), or ii) interfering with the interactions between tRNAs and ribosomes.

In this Perspective, we will focus on the second approach, repressing protein translation by targeting the tRNA–ribosome interaction. (Although the first approach is likely to be more effective against cancers [19], the details are beyond the scope of a short Perspective such as this.) For viral infections, this second approach may be effective thanks to a key molecule, Homoharringtonine (HHT), a cytotoxic plant alkaloid extracted from *Cephalotaxus* species [20]. HHT is referred to as omacetaxine mepesuccinate in its semi-synthetic form. HHT competes with the amino acid side chains of aminoacyl-tRNAs for binding to the A-site cleft of the ribosome. The competition may hinder the correct positioning of aminoacyl-tRNAs and prevent protein elongation [21–24]. Clearly, HHT would affect those proteins with short half-lives strongly as their amounts would decrease rapidly without replenishment [25,26].

HHT was approved by the FDA for treatment of chronic myeloid leukemia (CML) in 2012 [27]. Since then, several clinical trials on patients with various forms of leukemia have demonstrated the efficacy and safety of HHT in hematological malignancies. These include acute myeloid leukemia (AML [28]), *FLT3*-ITD AML (with *FLT3*-ITD mutations [29]), and higher risk-myelodysplastic syndromes or chronic myelomonocytic leukemia [30]. HHT is also effective in a subset of patients with chronic myeloid leukemia in the accelerated phase or blast phase, suggesting that HHT may work particularly well against very rapidly dividing cells [31]. In contrast, results of HHT in solid tumors were negative, likely because of the large percentages of slowly dividing cells in solid tumors [32,33].

## VIRUSES

Like cancer cells, virus-infected cells must make much larger quantities of proteins than normal. These cells are making viral particles rather than the proteins they require for themselves. Among the viruses, we shall focus mainly on the single-stranded RNA viruses (SS+ for short, + meaning positive strand), which have been extensively investigated. It has been shown that SS+ viruses can quickly take over translation in human cells. Within a few hours of infection, translation activities for host cells’ own needs decrease by more than 80% [34].

Because virus-infected human cells expend most of their effort on translating viral proteins, it would seem logical to repress protein translation as a means of repressing viral production. In other words, the attack would be on the cellular components, rather than on the viruses themselves. However, such an approach raises two questions.

### Why attack the cellular machineries assisting viruses, rather than attacking the viruses directly?

We should note that many research and development efforts are indeed devoted to this aspect of controlling viral infection. For example, favipiravir targeting RDRP (RNA-dependent RNA polymerase, which is specific to the virus) has been declared at least partially effective in treating patients. Nafamostat is also reported to be capable of preventing viruses from entering cells via the ACE2 receptor. Other nucleoside analogs, such as ribavirin and remdesivir, that interfere with viral replications are also being tested [35]. Unfortunately, when re-purposed for treating SS+ viral infections, most approved drugs have high IC_50_ (usually in the micromolar range), which is difficult to achieve in clinical applications [36]. Thus, many emerging SS+ viruses are still without effective antiviral drugs and re-purposing of other existing treatment strategies is urgently needed [36,37]. We suggest that the re-purposing should span a wider range than commonly attempted, for example, from cancer to viral infections.

### Would such an approach result in effective attack on infected cells while sparing uninfected cells, hence reducing undesirable cytotoxicity?

The short answer is ‘maybe’. Because the target is the aberrantly heightened translation activities in the infected cells, selective targeting is plausible. The issue has been addressed empirically. Since 2004 after a public health crisis similar to the current one subsided, the interference with the translation machinery has become an emerging approach in anti-virus studies [38,39]. In particular, HHT appears to be one of the most effective (if not the most effective) drugs against SS+ viruses in several studies. HHT inhibited Chikungunya virus infection very effectively and with minimal cytotoxicity at 1 μM concentration [40]. In *in vitro* screening based on 727 compounds from the existing library, HHT was found to be the strongest inhibitor of various SS+ viruses, with an IC_50_ as low as 12 nM [41]. Most recently, anti-viral activity of HHT was reported at nanomolar concentrations against porcine epidemic diarrhea virus and Echovirus 1 [42,43]. While these experimental studies suggest that HHT can be effective against many SS+ viruses, the mechanisms of effectiveness need to be understood (or at least postulated) as a basis for possible clinical applications. Two parallel mechanisms may operate side-by-side that make HHT a particularly effective drug against SS+ viruses.

The first is a general mechanism of translation repression that works in most cells. When the synthesis of new proteins is halted, those with a short half-life quickly become too few to meet the demand. In cancers such as AML, this mechanism is often cited as the reason for the efficacy of HHT whereby many transcription factors driving cancer progression are in short supply. Similarly, proteins that are needed in large quantities will quickly decrease below the threshold. For SS+ virus, structural proteins including the four main ones (S, M, E and N for spike, membrane, envelope and nucleocapsid proteins) are in this category. If there are no new structural proteins made, there will be no new viral particles.

The second mechanism is of particular interest. Many SS+ viruses have an unusual coding strategy by stringing many of their non-structural proteins (nsp) into a super-polypeptide, which is then cleaved into individual proteins. These super-proteins can be upward of 700 kda (∼8000 amino acids, or AAs, in length) in a size range where very few host proteins are found (Table 1 and Table S1). In discussing the mechanism of disrupting very large proteins, we shall consider the clinical dosage of HHT, which is usually at the nM level. In contrast, the concentration in laboratory experiments is usually several hundred-fold higher than in the clinical setting.

**Table 1.** Tissue expressions of the six largest human proteins.

	CDS			
Gene	length (nt)	Tissue expression		
TTN	107976	Heart (4E+09)	Small intestine (3E+07)	Fat (2E+07)
MUC16	43524	Fallopian tube (2E+06)	Lung (3E+05)	Liver (2E+05)
OBSCN	26772	Heart (4E+08)	Pancreas (5E+06)	Colon (2E+06)
SYNE1	26394	Fallopian tube (4E+07)	Brain (4E+07)	Stomach (2E+07)
NEB	25683	Heart (6E+06)	Prostate (5E+06)	Tonsil (3E+06)
SYNE2	20724	Testis (6E+07)	Placenta (4E+07)	Kidney (3E+07)

For each gene, the three tissues with the highest expression levels are shown. Numbers in the brackets indicate the normalized iBAQ values of protein expression (extracted from [44]).

At low (clinical) concentration, HHT disrupts protein elongation by preventing the incoming aminoacyl-tRNA from unloading its amino acid cargo [21]; thus, longer proteins are more likely to be aborted before completion. Assuming that HHT interferes with each step of peptide elongation by a probability p, the cumulative probability of completing the synthesis of a protein with k AAs would be (1-p)∧k ∼ e^−pk^. If a protein of 500 AAs in length has an 80% chance of surviving the HHT treatment, then one that is 8000 AAs long will be successfully translated only 4% of the time.

While the mechanism at high and low concentrations of HHT should not be different, quantitative differences could be mis-construed as qualitative in nature. At the high concentration commonly used in the laboratory experiments, p may be close to 1, especially near the translation start site. With high p value, the probability of translating beyond the first few AAs is small. This may be the reason for the common interpretation that HHT represses the ‘initial translation’ of proteins. This is discussed in more detail in the Supplementary data.

**Figure 1.**
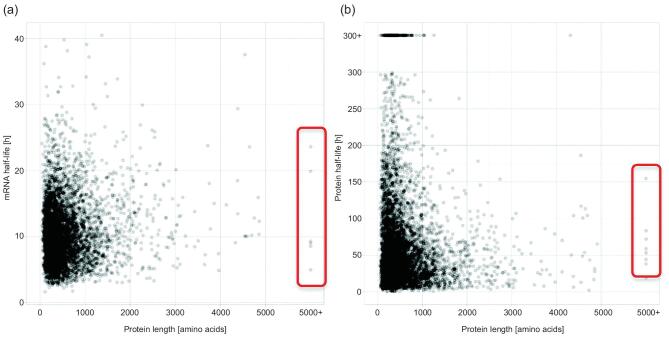
mRNA (a) and protein (b) half-lives of mammalian genes as a function of the protein size (extracted from [4]). The values of the largest proteins with >5000 amino acids are shown in the red-border boxes. In Table 1, we note that large proteins are often tissue-specific. Because the half-lives of these large proteins, at >50 hours, are not unusually short, halting the production of large proteins transiently when treating virus-infected cells should not have large cytotoxicity effects.

As the nsp super-protein of the virus is crucial for viral production, it offers an opportunity for virus-specific targeting. The question is whether, and how, the very large proteins in normal human cells could be similarly affected. Table 1 shows the six largest human proteins, all >7000 AAs in length (see [44]). Their expressions are mainly in the heart and reproductive tissues, although the fourth to sixth ranked proteins have a broader tissue distribution. Table S1 presents data on the 23 proteins that are >5000 AAs in length, some of which also have a broad tissue distribution. Fortunately, the mRNAs and polypeptides of these very large proteins do not have shorter half-lives than the genomic average, at >48 hours for the large proteins (see Fig. 1a, b). These general patterns suggest that targeting very large proteins may disrupt the viral production, with minimal effects on the functions of the normal cells.

In short, the viral coding strategy of producing one or two ‘super proteins’ is perhaps efficient but could also be the Achilles heel of the SS+ viruses. We hypothesize that this mechanism may play a role in the efficacy of HHT in suppressing viral production.

### Potential of HHT in reducing viral load in clinical settings

The hypothesis of the mechanisms of translation repression suggests potential application of HHT in treating viral infections in the current crisis. In particular, HHT, with IC_50_ = 12 nM *in vitro*, is known to be one of the most effective drugs at repressing translation, thus making it less of a challenge to sustain adequate concentration near the infection sites. The clinical dosages of HHT in fighting leukemia [27] seem quite adequate to treat viral infections *in vivo* [42], at roughly 0.05 mg/kg per day. Furthermore, in animal experiments (piglets infected with porcine epidemic diarrhea virus [42]), the treatment would yield detectable reductions in viral load in 3 days, while the leukemia treatment lasts for six cycles, each cycle being 14 days of treatment followed by 14 days of rest. Hence, the toxicity effect at the clinically effective dosage should be more manageable in treating viral infections than in treating cancers.

Although using HHT to treat SS+ viral infections in humans is a theoretical conjecture, it is, nevertheless, based on understanding of the underlying mechanisms. For possible clinical applications, we will add two more considerations and one caveat. First, one may imagine using higher doses in the early phases of treatment to stop production of structural proteins. This may lead to quick shedding of the viral load. Subsequently, one may switch to lower doses sufficient to abort the translation of the multi-nsp super-protein, thus persistently suppressing the re-emergence of viral production. Second, it may be possible to use a nebulizer to deliver HHT to the lung, assuming that it is the main infected organ. Nebulization is apparently being used in current clinical practices to treat SS+ viral infections. The drug may then spread to other organs via the blood circulation through the lungs, which would receive a higher dose than other tissues.

The caveat is that the proposal is about suppressing viral production and reducing the viral load. After tissues are damaged by the viral infection, either directly or indirectly via other infections or immune over-response, the treatment may or may not be too late. Furthermore, while the treatment strategy aims at viral suppression with minimal damage to the uninfected cells, the recovery of the infected cells after viral clearance will not be known until relevant clinical data are available. The caveat suggests that the proposed HHT treatment should start as soon as the symptom appears. In theory, the strategy should arrest the progression of infection at the stages where the immune system can function properly.

## CONCLUSION

HHT was identified about 50 years ago. Its mechanisms of action and safety are now well understood, leading to wide uses in treatment of cancer. It also inhibits SS+ viral replication at the nanomolar concentration, making clinical application achievable in patients. Furthermore, HHT targets the highly conserved translation machinery without unduly interfering with the immune system. By reducing the viral load, HHT may help the immune system to function properly under reduced stresses.

In this Perspective, we treat the urgent and pragmatic issues as theoretical problems. Many clinical phenomena are in this category. Cancers [10,13,45–48] and viral infections are two immediate examples.

## Supplementary Material

nwaa066_Supplemental_File
